# Exosomal transfer of tumor-associated macrophage-derived miR-21 confers cisplatin resistance in gastric cancer cells

**DOI:** 10.1186/s13046-017-0528-y

**Published:** 2017-04-13

**Authors:** Peiming Zheng, Lei Chen, Xiangliang Yuan, Qin Luo, Yi Liu, Guohua Xie, Yanhui Ma, Lisong Shen

**Affiliations:** 1 Department of Clinical Laboratory, Xinhua Hospital, Shanghai Jiao Tong University School of Medicine, Shanghai, 200092 China; 2 Department of General Surgery, Xinhua Hospital, Shanghai Jiao Tong University School of Medicine, Shanghai, 200092 China

**Keywords:** Cisplatin resistance, Tumor-associated macrophages, Exosome, miR-21, Gastric cancer

## Abstract

**Background:**

Cisplatin-based chemotherapy is frequently used to treat advanced gastric cancer (GC). However, the resistance often occurs with the mechanisms being not well understood. Recently, emerging evidence indicates that tumor-associated macrophages (TAMs) play an important role in chemoresistance of cancer. As the important mediators in intercellular communications, exosomes secreted by host cells mediate the exchange of genetic materials and proteins to be involved in tumor aggressiveness. The aim of the study was to investigate whether exosomes derived from TAMs mediate cisplatin resistance in gastric cancer.

**Methods:**

M2 polarized macrophages were obtained from mouse bone marrow or human PBMCs stimulated with IL-4 and IL-13. Exosomes isolated from M2 macrophages culture medium were characterized, and miRNA expression profiles of M2 derived exosomes (M2-exos) were analyzed using miRNA microarray. In vitro cell coculture was further conducted to investigate M2-exos mediated crosstalk between TAMs and tumor cells. Moreover, the in vivo experiments were performed using a subcutaneous transplantation tumor model in athymic nude mice.

**Results:**

In this study, we showed that M2 polarized macrophages promoted cisplatin (DDP) resistance in gastric cancer cells and exosomes derived from M2 macrophages (M2-exos) are involved in mediating the resistance to DDP. Using miRNA profiles assay, we identify significantly higher levels of microRNA-21 (miR21) isomiRNAs in exosomes and cell lysate isolated from M2 polarized macrophage. Functional studies revealed that exosomal miR-21 can be directly transferred from macrophages to the gastric cancer cells, where it suppresses cell apoptosis and enhances activation of PI3K/AKT signaling pathway by down-regulation of PTEN.

**Conclusions:**

Our findings suggest that exosomal transfer of tumor-associated macrophages derived miR-21 confer DDP resistance in gastric cancer, and targeting exosome communication may be a promising new therapeutic strategy for gastric cancer patients.

**Electronic supplementary material:**

The online version of this article (doi:10.1186/s13046-017-0528-y) contains supplementary material, which is available to authorized users.

## Background

Gastric cancer is currently the fourth most common malignancy and the third leading cause of cancer-related deaths worldwide [1]. The incidence and mortality of gastric cancer are the highest in East Asia (particularly in Korea, Mongolia, Japan, and China), and it has become the second most lethal cancer in China [2]. The poor prognosis of this cancer resulted from late detection, aggressive characteristics and poor response to available therapies. Although combined chemotherapy pre- and post-operation has increased patient survival rates, the development of drug resistance is still the most significant obstacles to effective chemotherapy [3]. Cisplatin remains to be a primary chemotherapeutic drug for gastric cancer patients, especially for advanced stage ones. However, resistance often occurs with the mechanisms being not well understood, which results in relapse of cancer and poor survival. The elucidation of molecular mechanisms to cisplatin resistance is important for improving gastric cancer survival.

It is well known that the tumor microenvironment comprises a variety of nonmalignant stromal cells that evolves with and provides support to tumor cells during the tumor progression [4, 5]. Among them, tumor-associated macrophages (TAMs) are the major components and play a pivotal role in tumor growth, angiogenesis, metastasis and therapy resistance [6–8]. Macrophages are heterogeneous cells that undergo different functional reprogramming in response to various stimulating signals. M1- and M2-polarized macrophages, activated by IFNγ with LPS and IL-4 with IL-13 respectively, are extremes of a broad range of functional states [9–11]. In most solid tumor, TAMs are typically a macrophage subpopulation with M2 phenotype and a positive correlation between TAM density and poor prognosis has been proved in several types of cancer [7, 12], including gastric cancer [13, 14]. Increasing evidence has shown that TAM regulates therapeutic responses of cancer cell and immunotherapy targeting TAM maybe an innovative combination therapy designed to cure cancer [15, 16]. Nevertheless, the detailed interaction between anticancer therapies with TAM remains unclear.

More recent studies have demonstrated that cells can communicate with neighboring or distant cells through the secretion of exosomes. Exosomes are generated from multivesicular bodies (MVBs) and are secreted into the extracellular space through fuse with the plasma membrane. These vesicles range in size from 50 to 100 nm containing proteins, lipids, mRNA, and are enriched with miRNA [17–19]. Several studies have shown that many types of cells can release exosomes and exosomal transmission among tumor microenvironment cells modulates therapeutic resistance of cancer cells [20–22]. MicroRNAs are small, noncoding RNAs that control the expression of multiple target genes at the posttranscriptional level. Interestingly, exosomal miRNAs are more stable and the transfer of miRNA by exosomes contributes to the development of chemoresistance in multiple tumourtypes [23, 24]. However, the miRNA signatures of TAM-derived exosomes have not been identified and whether these exosomal miRNAs are involved in chemoresistance in gastric cancer remain unknown.

In this study, we first construct the TAM-like M2 polarized macrophages activated by IL-4 with IL-13 and show that macrophage-derived exosomes can be ingested by gastric cancer cells, reducing the chemotherapy sensitivity to cisplatin. MicroRNA expression profiles using miRNA array reveals that miR-21a-5p is the most abundant in M2 macrophage-derived exosomes. Further investigation demonstrates that miR-21 can be directly transferred, through exosomes, from TAM to gastric cancer cells, and regulates the chemotherapy resistance of these cells. Our studies not only reveal a novel communication mechanism between TAM and gastric cancer cell, but also may provide a promising new therapeutic target for gastric cancer patients.

## Methods

### Cell culture and treatment

The gastric cancer cell line MFC,MGC-803 were purchased from the Chinese Academy of Sciences Cell Bank of Type Culture Collection. Murine bone marrow–derived macrophages (BMDM) were isolated and activated as previously described [25]. Briefly, bone marrow cells from femur of C57BL/6 male mice were isolated and cultured for 7 days in DMEM:F12 (Gibco, Life technologies, USA) supplemented with 10% FBS (Gibco, USA) and 50 ng/ml M-CSF (R&D Systems, USA). Media was changed every 3 days and contaminating nonadherent cells are eliminated and adherent cells are harvested for further stimulated. The cells were incubated for 48 h with 20 ng/ml IL-4 plus 20 ng/ml IL-13 (PeproTech, USA) to achieve the M2 polarized macrophages. For in vitro differentiation of human monocytes into macrophages, monocytes were isolated by negative selection from PBMCs using magnetic beads (MiltenyiBiotec), then isolated cells were subsequently cultured in in RPMI1640 supplemented with 10% FBS (Gibco) and 100 IU/ml rhM-CSF for 7 days. The polarization of the resulting monocyte-derived macrophages was obtained as above described. The other cells were cultured at 37 °C with 5% CO2 in DMEM containing 10% FBS supplemented with 100 U/mL penicillin and 100 μg/ml streptomycin (Gibco). For the co-culture experiment, Macrophage were grown on the 0.4um pore size transwell insert (Corning) and the GC cells were grown in the bottom well of the transwell chamber.

### Flow cytometry

The M2 polarized macrophage were trypsinized and washed twice in 1 × PBS, after resuspended in 100ul 1 × PBS, fluorochrome-conjugated antibodies against F4/80, CD11b, CD206, CD86, CD163, CD68, CD80 or their respective isotype controls were added and stained for 30 min at 4 °C. Following washed twice in 1 × PBS, labeled cells were analyzed by flow cytometry on a FACS Canto II flow cytometer (BD Biosciences) and analyzed with FlowJo software (Tree Star). All antibodies used for FACS are listed in (Additional file 1: Table S1).

### Apoptosis assay

Apoptosis was measured using the FITC Annexin V Apoptosis Detection Kit I (BD Pharmagen, USA) following the manufacturer’s protocol. In brief, cells were washed twice with cold PBS and then resuspended in 100 μl of 1X Binding Buffer, then add 5 μl of FITC Annexin V and 5 μlpropidium iodide (PI) for 15 min at room temperature in the dark. After incubation 400 μl of 1X Binding Buffer were added to each tube and analyzed by FACS Canto II flow cytometry (BD Biosciences).

### Exosome isolation and analysis

Macrophages were incubated for 48 h in DMEM:F12 medium with 10% exosome-free FBS. This conditioned medium was collected and exosomes were isolated using Exosome Precipitation Solution (System Biosciences, USA). Identification of exosomes was processed according to the protocol described in Exosome Antibody Array (System Biosciences).

For exosome uptake experiments, exosome preparations were labeled with PKH67 Fluorescent Cell Linker Kits (Sigma-Aldrich) according to the manufacturer’s instructions, followed by washing through Exosome Spin Columns (MW3000) (Invitrogen, USA) to remove excess dye. Next, exosomes were incubated with gastric cancer cells and examined under a SP5 confocal microscope (Leica, USA).

### Transmission electron microscopy (TEM)

For TEM, 10 μl of exosome suspension were absorbed onto carbon-coated cooper grids (200 mesh) for 1 min. Samples were washed with double-distilled water and negatively stained with 2% uranyl acetate solution for 1 min. After air dry, the samples were visualized at 87000x in a Phillips Tecnai transmission electron microscope at 80 kV.

### MicroRNA microarray

Exosome pellets from 10 ml supernatant of M2 polarized macrophages were collected and homogenized in Trizol (Invitrogen). Total RNA was quantified with a NanoDrop 2000c spectrophotometer (Thermo Scientific, USA) and its quality was assessed by capillary electrophoresis on an Agilent 2100 Bioanalyzer (Agilent Technologies, CA). The miRNA microarray analysis was performed by Shanghai Biotechnology Corporation

### Transfection of miRNA mimics and negative control

For in vitro transfection of miRNA, Cy3-labeled miR-21 mimics and negative control (GenePharma, China) were transfected using Lipofectamine 3000 (Life Technologies), according to the manufacturer’s instructions. After 24 h of transfection cells were collected and used for further analysis

### In vitro detection of miR-21 transfer

To further observe the transfer of miRNA, Exosomes prepared from M2 macrophages transfected with Cy3-labelled miR-21 or without transfection (ctrl) were added to MFC cell cultures. MFC cells were were fixed in 4% PFA, treated with 0.3% Triton X-100, blocked with 3% BSA at 37 °C. After being washed with PBS, Cellular F-actin was visualized by staining with Alexa 488 phalloidin (LifeTechnologies, USA) according to the manufacturer’s guidelines. Cells were mounted with ProLong® Gold antifade Reagent with DAPI (LifeTechnologies, USA). Images were captured using Leica SP5 Laser scanning confocal microscope.

### Cell viability and adhesion-dependent colony formation assay

Gastric cancer cells were seeded in 96-well plate at 1500–3000 cells per well and incubated with DDP for 24–72 h, cell viability was detected with the Cell counting Kit-8 (Dojindo Laboratories, Japan). The optical density at 450 nm was measured on a multiwall plate reader (FLX800, Bio-TEK). Transfected gastric cancer cells were plated in 60-mm dishes at a density of 2 × 10^3^ cells per well for adhesion-dependent colony formation assay. DDP was added to the culture medium at a final concentration of 5uM. Culture medium that contained DDP was changed every 3–4 days. Then, 3–4 weeks later, the remaining colonies were fixed with 4% paraformaldehyde and dyed with crystal violet. The colonies were counted according to the defined colony size.

### RNA extraction and quantitative real-time PCR

Total RNA was extracted using TRIzol reagent (Invitrogen, USA) according to the manufacturer’s instructions. The concentration and quality of the total RNA were assessed with Nanodrop Spectrophotometer (Thermo Fisher Scientific, USA). For the mRNA expression analysis, reverse transcription was performed using PrimeScript RT master mix (TaKaRa, Japan). For miRNA expression analysis, total RNA was first reverse transcribed using Mir-X™ miRNA First-Strand Synthesis Kit (TaKaRa, Japan). Quantitative real-time PCR analysis was performed in triplicate on 7900 HT Real-Time PCR System (Applied Biosystems, USA) using SYBR Premix Ex Taq (TaKaRa, Japan) and the expression levels of GAPDH or U6 was used as endogenous control. The 5′primer used for miR-21 is TAGCTTATCAGACTGATGTTGA, the mRQ3′ primer and U6 primers are supplied with the kit. Results were analyzed using the 2^–ΔΔct^ calculation method. Other primers sequences of mentioned genes are described in (Additional file 2: Table S2).

### Western blot

The cells were lysed in equal volumes of ice cold lysis buffer and a protease inhibitor cocktail. Cell lysate were separated by SDS-PAGE and then transferred to a 0.2-μm PVDF membrane (Bio- Rad, USA). After blocking with Odyssey Blocking Buffer (Li-COR Biosciences, USA), the membrane was incubated with primary antibody (1:1000) at 4 °C overnight, followed by incubation with IRDye 800CW or 680 secondary antibodies (1:5000, LI-COR Biosciences, USA). GAPDH was used as endogenous control. The Odyssey Infrared Imaging System was used to visualize targeted protein bands. All antibodies used for western blot are listed in (Additional file 1: Table S1).

### In vivo xenograft and treatment experiments

For in vivo studies, 4–6 week old male athymicC57 nude mice were purchased from Shanghai Laboratory Animal Center of China. MFC cells (3× 10^5^ cells in 200ul PBS) pretreated with or without M2-Exos were subcutaneously injected into the nude mice to establish tumors. Another group received subcutaneous injections of the same MFC cells transfected with miR-21 or miR-NC. After 10 days, 10 mg/kg DDP or PBSwas injected intraperitoneally. M2-Exos or miR-21 were injected twice intratumorally before the start of DDP treatment. The mice were examined every 2 days and sacrificed 6–7 days after DDP treatment. The tumor sizes were measured using digital caliper and tumor volume was calculated with the following formula: volume = 0.5 × width^2^ × length. All animal procedures were carried out with the approval of the Institutional Committee of Shanghai Jiao Tong University School of Medicine for Animal Research.

### Statistical analysis

Statistical significance between groups was determined by a two-tailed Student’s t-test and a one-way ANOVA test. Differences were considered to be significant when *P* < 0.05. All statistical data were displayed as means ± standard deviation (SD) and analyzed for statistical significance with GraphPad Prism 5.0 for Windows (GraphPad Software, USA).

## Results

### M2 polarized macrophages induces the resistance of gastric cancer cells to cisplatin

**Fig. 1:**
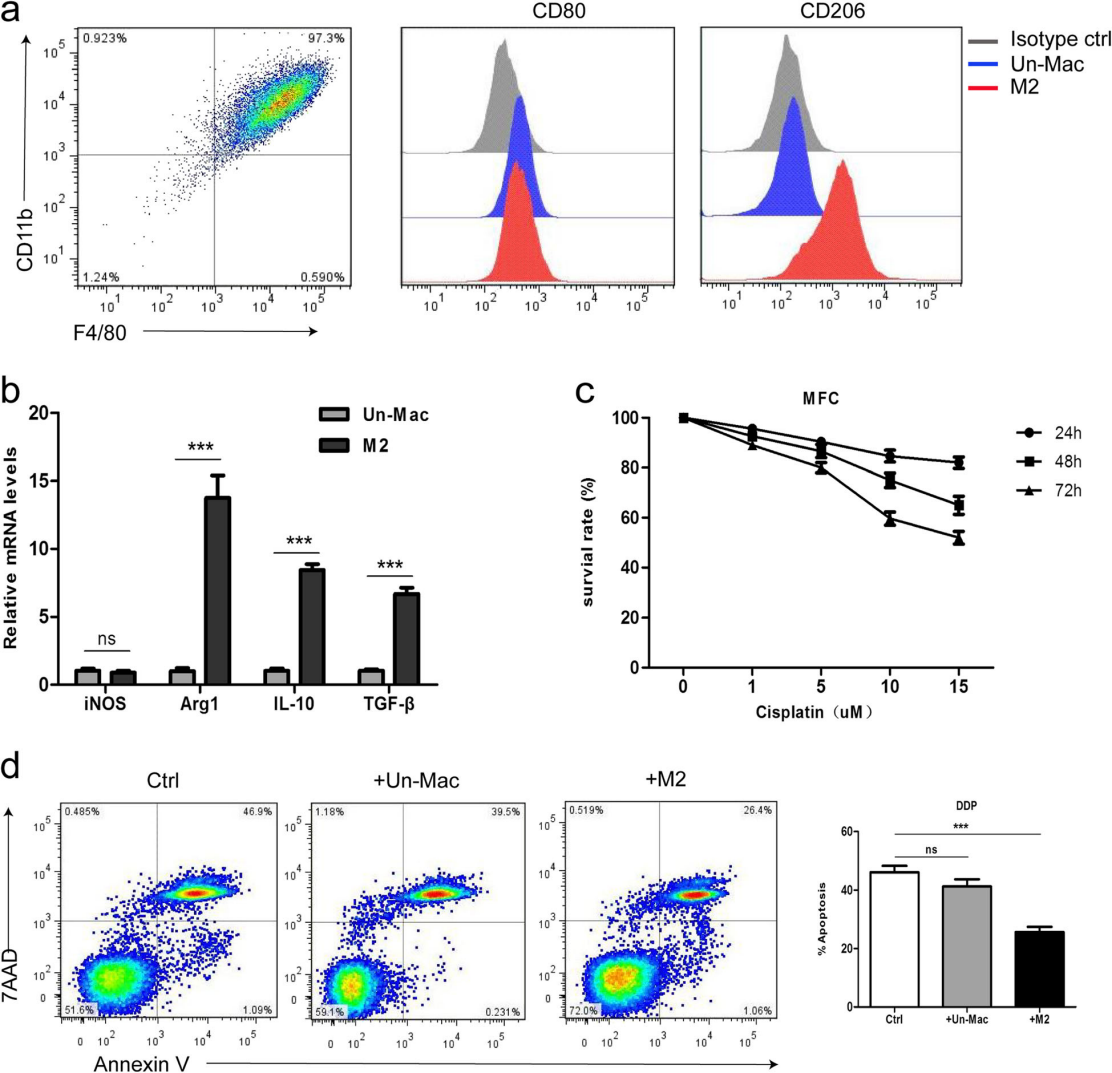
Co-cultivation with M2 polarized macrophages enhances the resistance of MFC cells to cisplatin. **a** FCM identification of M2 polarized macrophages derived from murine bone marrow stimulated with IL-4 and IL-13, CD206 was a specific marker for M2. (Un-Mac, unactivated macrophages; M2, macrophages activated by IL-4 and IL-13). **b** qRT-PCR detection of iNOS, Arg1, IL-10, TGF-β mRNA expression in unactivated and M2 polarized macrophages, GAPDH was assayed as an internal control. (ns *p* > 0.05, *** *p* < 0.001). **c** Cell viability assay of MFC treated with various concentrations of DDP for 24 h, 48 h, and 72 h. **d** Flow cytometric analyses of apoptotic cells. MFC cells were cultured alone or co-cultured with unactivated (Un-Mac) or M2 polarized macrophages, and then exposed to DDP for 48 h. The quantitative data are presented as the mean ± SD of triplicate experiments. (ns *p* > 0.05, *** *p* < 0.001)

Previous study and our unpublished data indicated that TAMs infiltrated in gastric cancer are primarily M2 macrophages. To determine the functional biology of polarized macrophages, we generated M2-polarized macrophages in vitro from mouse bone marrow cells (MBMCs) cultured in the presence of M-CSF with IL-4/IL-13. The M2 polarized macrophages were identified through flow cytometry analysis for expression of surface antigens, including CD11b, F4/80, CD206, and CD86 (Fig. 1a). In addition, specific gene expression was evaluated using quantitative RT-PCR (Fig. 1b). The expression of prototypical M2 markers (CD206, Arg1, IL-10, TGF-β) was increased, indicating that we successfully derived M2 macrophages from MBMC.A similar observation regarding the markers was made in human M2-polarized macrophages from human peripheral blood monocytes (PBMCs) (Additional file 3: Figure S1a).

In order to determine the optimal dosage levels of cisplatin (DDP), the gastric cancer cell lines MFC and MGC-803 were treated with various concentrations of DDP for 24, 48, and 72 h. CCK8 assay showed that the cell viability was gradually decline along with prolonged time and increasing concentration (Fig. 1c and Additional file 3: Figure S1b). Based on this observation, the doses used in the drug resistance test were as follows: MFC (15uM, 48 h), MGC-803 (7uM, 48 h). To determine whether M2 polarized macrophages confers chemoresistance in gastric cancer cells, we co-cultured gastric cancer cells with M2 polarized macrophages in a transwell insert, which prevents direct cell-cell contact. Then the gastric cancer cells were treated with DDP for 48 h, and the results showed a significant decrease of apoptosis rate in cells co-cultured with M2 polarized macrophages compared with those co-cultured with unactivated macrophages or normal control cells (Fig. 1d and Additional file 3: Figure S1c). Taken together, these data indicated that M2 polarized macrophages prompted the development of resistance to DDP in gastric cancer cells.

### Exosomes derived from M2 polarized macrophages can be ingested by gastric cancer cells

**Fig. 2:**
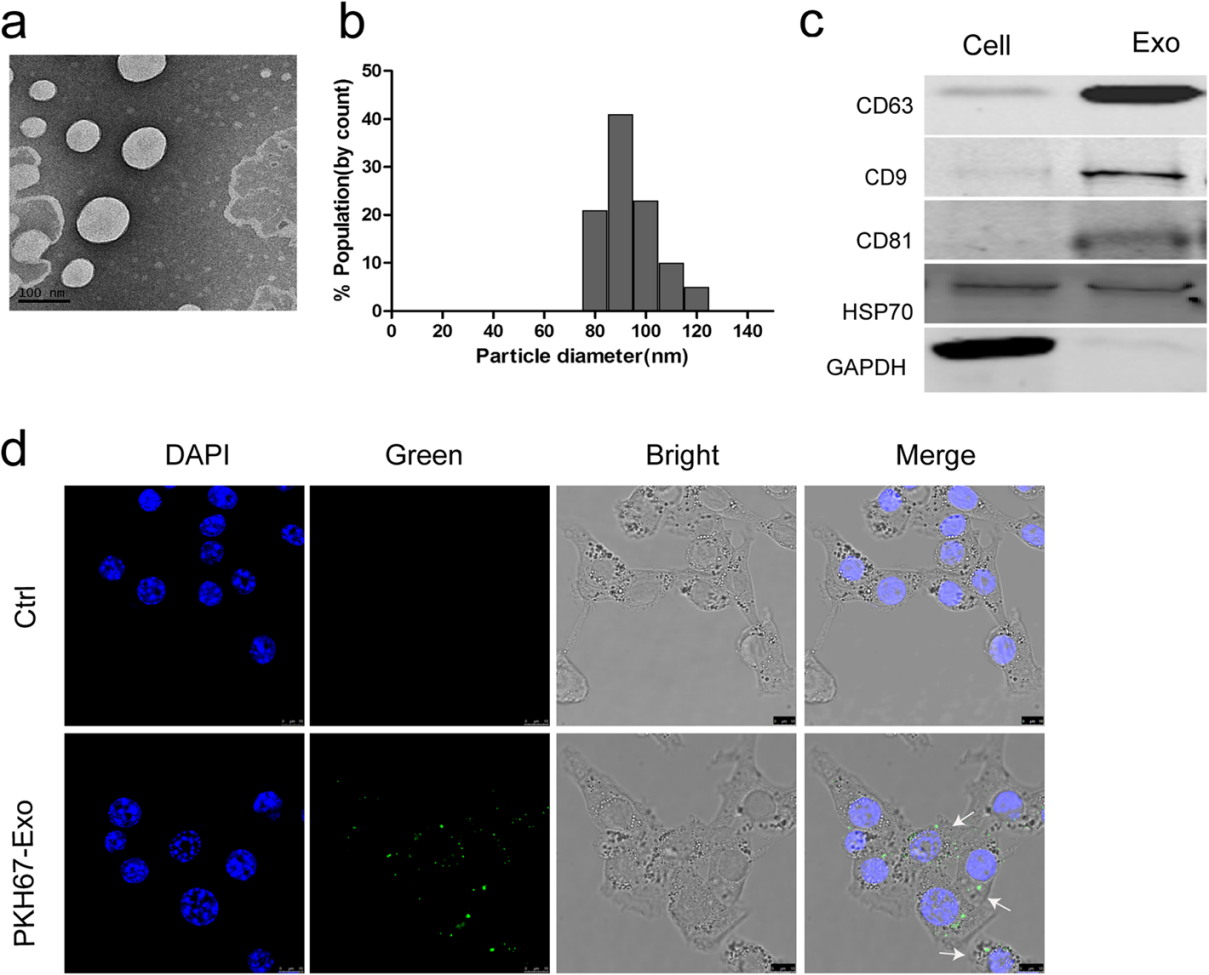
Exosomes derived from M2 polarized macrophages can be internalized by gastric cancer cells. **a** Representative transmission electron microscopy image of M2-derived exosomes (scale bar, 100 nm). **b** Histogram showing the particle diameter (nm) of the purified exosomes. **c** Exosomal markers (CD63, CD9, CD81, HSP70) were analyzed in M2 cellular protein and corresponding exosomes using western blotting (GAPDH was used as an internal reference). **d** The uptake of the PKH67 labelled M2-Exos was evident in MFCcells after 12 h of incubation. No stain was observed in the negative control condition. (scale bar, 10 um)

Previous studies have demonstrated that exosomes may regulate therapy resistance through transfer of functional small RNAs or proteins. To confirm whether M2 polarized macrophages-derived exosomes (M2-exos) play a pivotal role in the development of resistance of gastric cancer, we first isolated exosomes from the conditioned medium of M2 macrophages. The purified exosomes displayed as small round vesicles with a diameter ranging from 80 to120 nm, expressed the exosomalmarkers CD9, CD63 and CD81 (Fig. 2a, b, c).

To examine whether M2-exos can be taken up by gastric cancer cell, MFC cells were incubated with PKH67-labeled exosomes that were isolated from conditioned medium of M2 macrophages. Examination using confocal microscopy confirmed the uptake of PKH67-labeled exosomes by MFC cells (Fig. 2d), as evidenced by localization of green fluorescence and DAPI.

### M2-exos induce the resistance of gastric cancer cells to DDP in vitro and in vivo

**Fig. 3:**
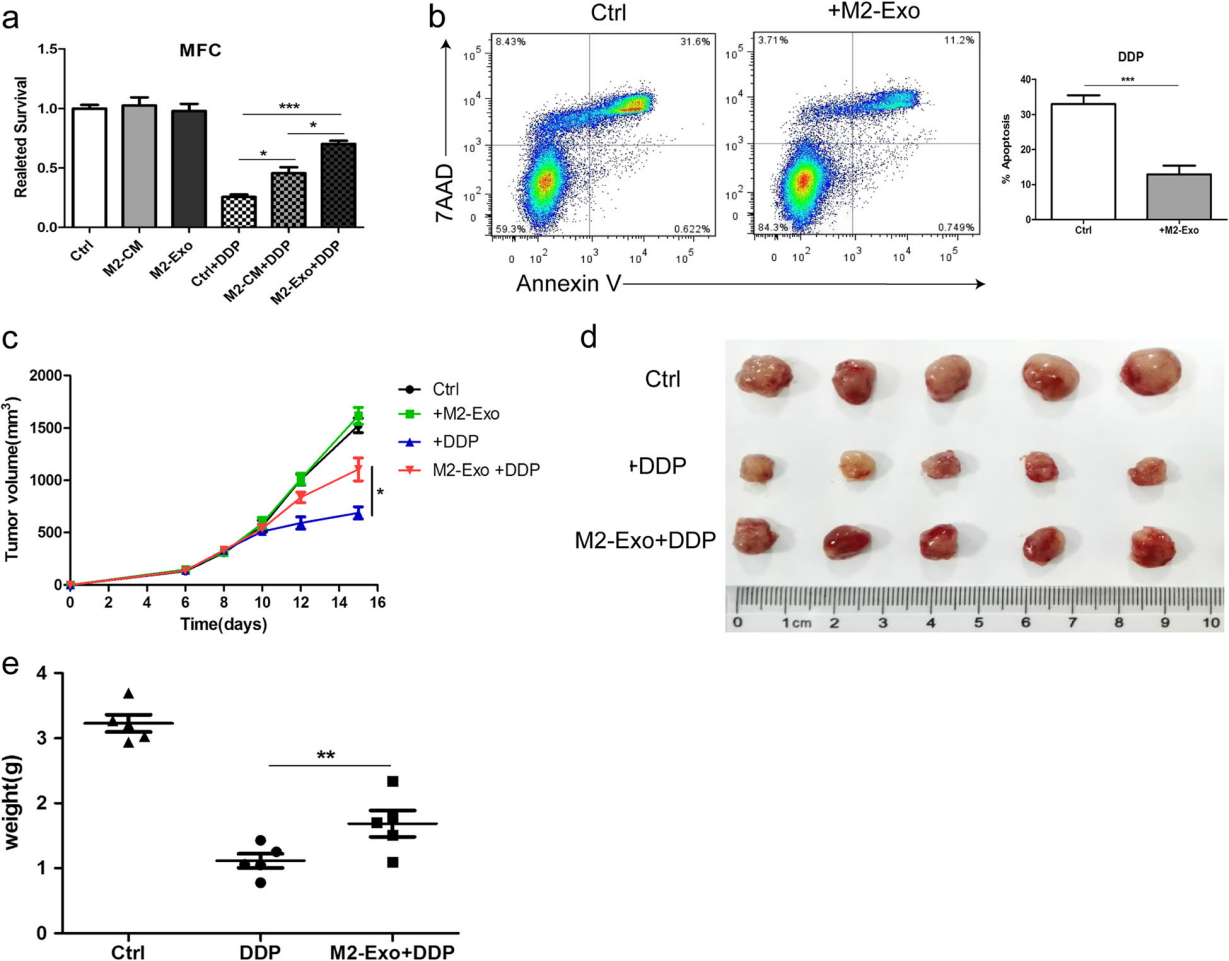
M2-exos induce the resistance of MFC cells to DDP in vitro and in vivo. **a** Cell viability was assessed by CCK-8 assays. M2-derived conditioned medium (CM) or exosomes (M2-Exo) attenuated DDP-induced cell suppression. MFC cells were pretreated with M2-derived CM or exosomes, and then exposed to DDP for 48 h. **b** Flow cytometric analyses of apoptotic cells. MFC cells were exposed to DDP alone (ctrl) or DDP and M2-Exo for 48 h. The quantitative data are presented as the mean ± SD of triplicate experiments. (*** *p* < 0.001). **c** Tumor growth curves in nude mice inoculated with MFC cells that were pretreated with or without M2-Exo.DDP or PBS (ctrl) treatment was initiated 10 days after subcutaneous injections of tumor cells. (*n* = 5, * *p* < 0.05). **d** The size of tumors at the end of the experiment from mice treated with PBS (ctrl), DDP, M2-Exo + DDP. (*n* = 5). **e** The mean weight of tumors from mice shown in (d). (*n* = 5, ** *p* < 0.01)

To further investigate the functional roles of M2-exos in the resistance of gastric cancer cells to DDP, MFC cells were incubated with conditioned medium (CM) or exosomes purified from M2 macrophages for 48 h. Then the cells were treated with 15uM DDP for another 48 h. CCK8 assay showed that co-cultured with M2-exos can significantly reduce the chemotherapy sensitivity of MFC cells and improve their relative survival rate (Fig. 3a). In addition, MFC cells co-treatment with M2-exos had a lower rate of apoptosis compared with normal treated cells (Fig. 3b). A similar phenomenon of chemoresistance was observed in human GC cell MGC-803 (Additional file 4: Figure S2a and S2b), confirming a common function of M2-exos in human and mouse models.

To demonstrate the role of M2-exos in the development of chemoresistance in MFC cells in vivo, MFC cells pretreated with M2-exos were injected subcutaneously into male nude mice, followed by an optimal dose of DDP treatment. MFC cells alone were injected as a negative control. We found that M2-exos significantly inhibited the chemotherapeutic effect of DDP, while M2-exos had minimal effect on the tumor growth (Fig. 3c). The final tumor size in M2-exos group was 1104 ± 92.2 mm^3^ after DDP treatment, which was significantly larger than that in DDP alone group (687 ± 78.3 mm^3^) (Fig. 3d). In addition, the mean tumor weight in M2-exos group was about 1.5 times heavier than that in DDP alone group (Fig. 3e). Taken together, these results suggested that M2-exos could induce the resistance of gastric cancer cells to DDP both in vitro and in vivo.

### Functional miR-21 is transferred from macrophages to gastric cancer cells through M2-exos

**Fig. 4:**
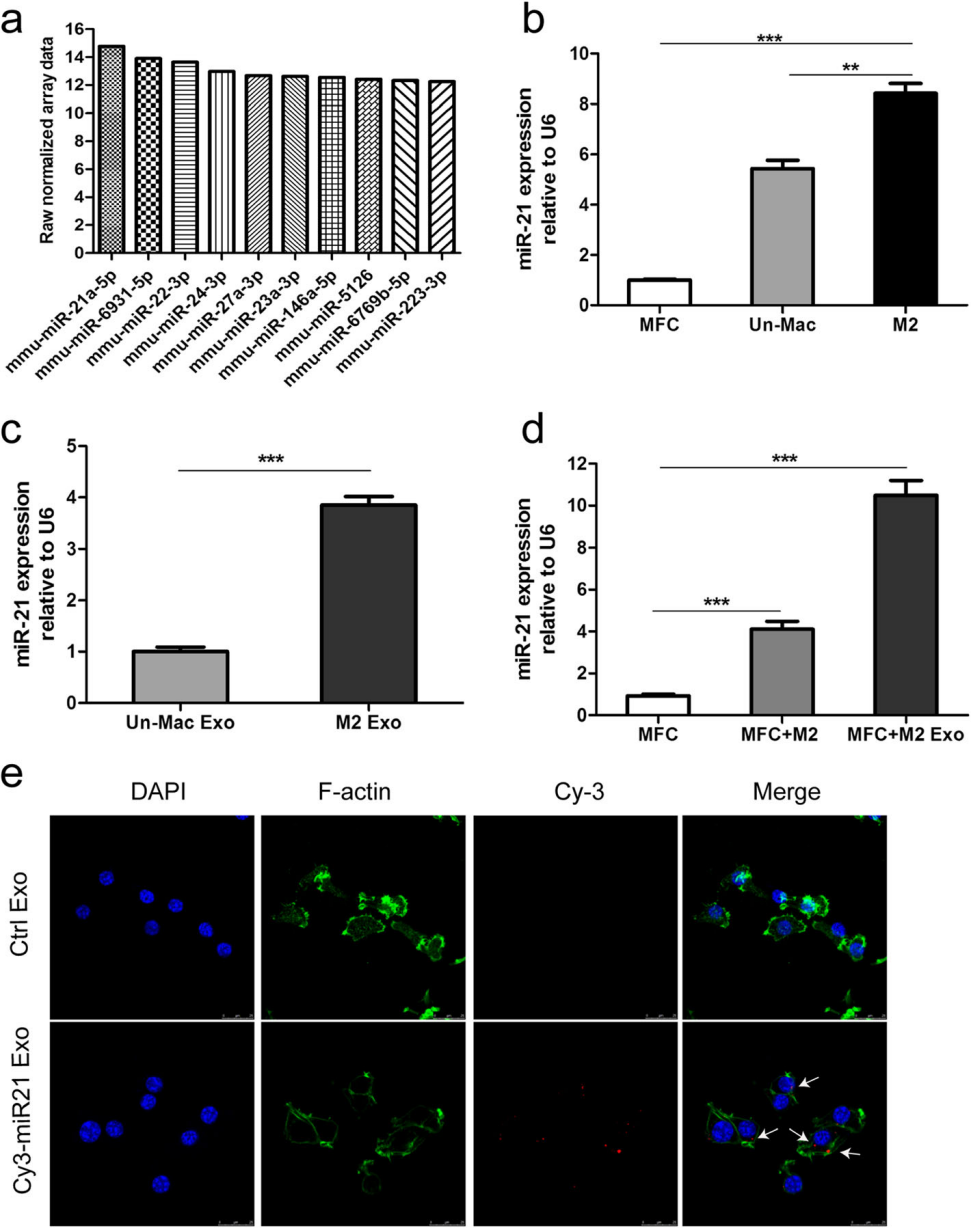
Exosomal transfer of miR-21 from M2 macrophages to gastric cancer cells. **a** The top 10 most abundant miRNAs in M2-Exo are listed, and miR-21 was the most abundant. **b** qRT-PCR detection of miR-21 in MFC, unactivated macrophages (Un-Mac) and M2 polarized macrophages (M2). (**p < 0.01, *** *p* < 0.001). **c** qRT-PCR detection of miR-21 in exosomes derived from unactivated macrophages (Un-Mac) and M2 polarized macrophages (M2). (*** *p* < 0.001). **d** qRT-PCR detection of miR-21 in MFC cultured alone (ctrl) or co-cultured with M2 macrophages or M2-Exo. (*** *p* < 0.001). **e** Exosomes prepared from M2 macrophages transfected with Cy3-labelled miR-21 or without transfection (ctrl) were added to MFC cell cultures. MFC cells were fixed and stained with DAPI and Alexa 488 phalloidin. The fluorescence signals in MFC cells were detected by SP5 confocal microscope. (scale bar, 25um)

Emerging evidence suggests that miRNAs which involved in cell-cell communication are frequently encapsulated in exosomes, and implement their biological functions in the recipient cells. To explore the mechanisms by which M2-exos conferred chemoresistance in gastric cancer, we generated miRNA profiles of M2-exos by miRNA microarray analysis. Among the miRNAs that were identified in M2-exos, miR-21 was the most abundant (Fig. 4a). Additionally, previous studies have demonstrated that miR-21 was involved in cancer chemoresistance; therefore, miR-21 was chose for further research. Using qRT-PCR, we confirmed that the miR-21 expression was much higher in macrophages as compared to MFC cells, expecially in M2 polarized macrophages (Fig. 4b). In addition, M2-exos contained higher levels of miR-21 than those from unactivated macrophages (Fig. 4c). Remarkably, the intracellular miR-21 levels in MFC cells was obviously increased after co-cultured with M2 polarized macrophages or M2-exos (Fig. 4d).

To further determine whether the elevated miR-21 level in MFC cells was resulted from directly shuttled from M2 macrophages to gastric cancer cells via exosomes, but not the tumor cells themselves. M2 polarized macrophages were transiently transfected with either Cy3-labeled miR-21 or negative control. We then isolated exosomes from conditioned media collected from the above macrophages. The purified exosomes were then added to gastric cancer cells culture medium. Using confocal microscopy, Cy3 labeled signals were detected in the gastric cancer cells incubated with exosomes isolated from Cy3-labeled miR-21 transfected cells but not in those incubated with exosomes from mock-transfected cells (Fig. 4e). In summary, these data suggest that M2 macrophage-derived exosomes mediate miR-21 shuttling.

### miR-21 reduces chemosensitivity and apoptosis in gastric cancer cells

**Fig. 5:**
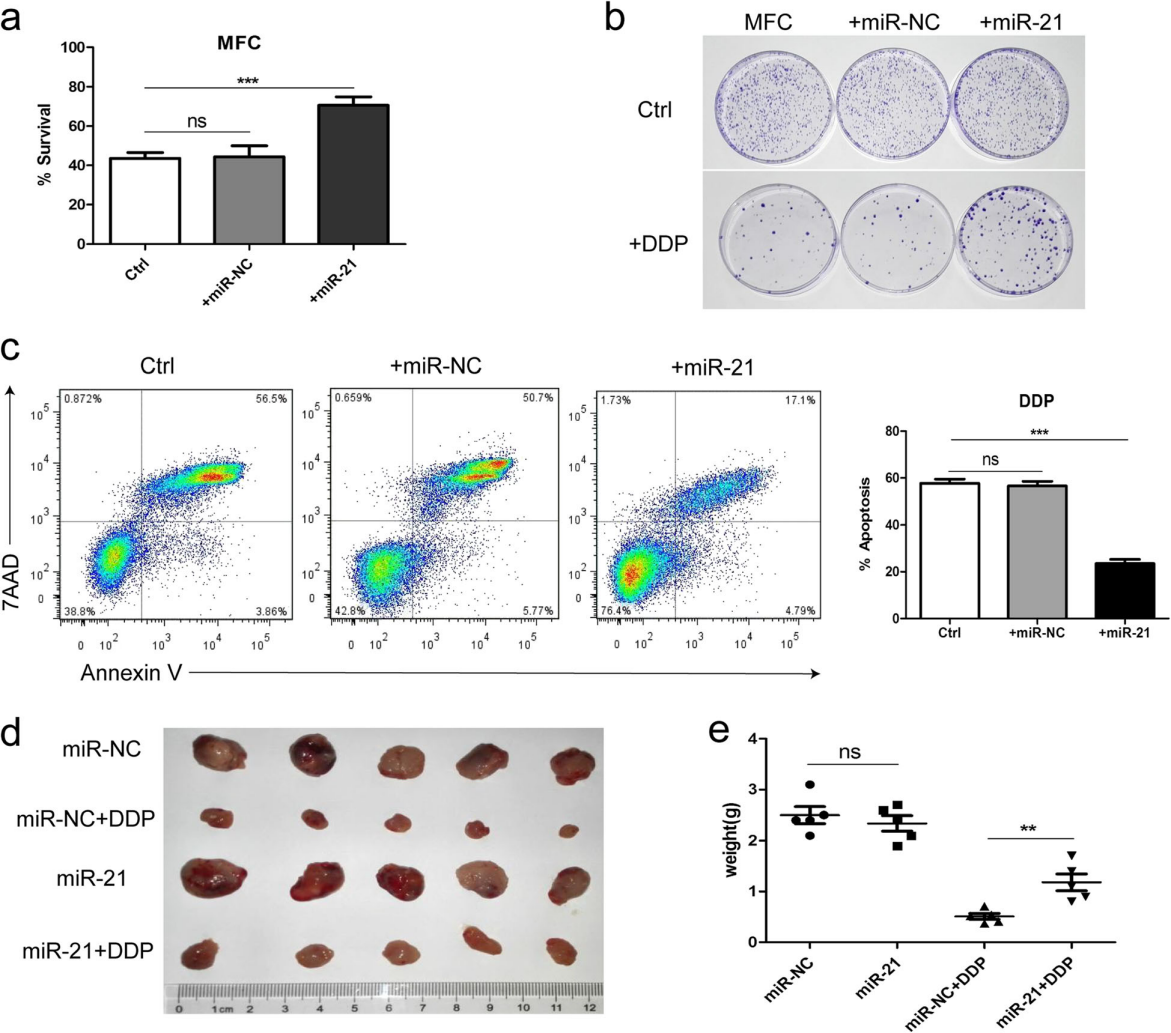
miR-21 induces the resistance of MFC cells to DDP in vitro and in vivo. **a** Cell viability assay of MFC cells treated with DDP after transfected with miR-21 mimics or miR-NC or without transfection (ctrl). **b** Colony formation assay of MFC cells transfected with miR-21 mimics or miR-NC or without transfection (up), and exposed to DDP (below). **c** Cell apoptosis assay of MFC cells treated with DDP after transfected with miR-21 mimics or miR-NC or without transfection (ctrl). The quantitative data are presented as the mean ± SD of triplicate experiments. (ns *p* > 0.05,*** *p* < 0.001). **d** and **e** The size and mean weight of tumors at the end of the experiment from mice inoculated with MFC cells transfected with miR-21 mimics or miR-NC. DDP or PBS (ctrl) treatment was initiated 10 days after subcutaneous injections of tumor cells. (*n* = 5, ns *p* > 0.05, ** *p* < 0.01)

To determine whether miR-21 can induce the chemoresistance in gastric cancer cells, we first examined the effects of miR-21 on gastric cancer cell chemosensitivity by directly transfecting miR-21 mimics or negative control into MFC or MGC-803 cells, and then treated them with DDP. The expression level of miR-21 in the transfected cells was increased markedly than did the mock transfectants (Additional file 5: Figure S3a). Cell viability was determined by using CCK-8 assay kit. Indeed, the cells transfected with the miR-21 mimics showed significantly lower chemosensitivity as compared to mock transfected cells (Fig. 5a and Additional file 5: Figure S3b). We further determined chemotherapy-induced apoptosis in gastric cancer cells transfected with miR-21 mimics or miR-NC. The apoptotic rate was analyzed by using FITC Annexin V apoptosis detection kit. A lower rate of apoptosis was observed in gastric cancer cells transfected with miR-21 mimics as compared to mock transfected cells (Fig. 5c and Additional file 5: Figure S3c). In addition, cell colony formation assay also demonstrated that miR-21 conferred chemoresistance in MFC cells (Fig. 5b).

To further investigate the functional roles of miR-21 in the chemoresistance of gastric cancer cells in vivo. MFC cells transfected with miR-21 mimics were injected subcutaneously into male nude mice, followed by an optimal dose of DDP treatment intraperitoneally. MFC cells transfected with miR-NC were injected as a negative control. We found that the final tumor size in miR-21 transfectant group was significantly larger and heavier than that in mock transfectant group (Fig. 5d and e), indicating that miR-21 significantly inhibited the chemotherapeutic effect of DDP. Taken together, these results suggested that miR-21 reduced chemosensitivity and apoptosis in gastric cancer cells.

### miR-21 shows no effect on ABC transporter genes in gastric cancer cells

The ABC (ATP binding cassette) family of membrane transport proteins, comprises of seven subfamilies ranging from A to G, play an important role in cancer chemoresistance. Certain ABC transporters couple the hydrolysis of ATP to move drug and xenobiotics unidirectionally out of cells, thereby effecting drug resistance [26]. In here, in order to determine whether miR-21 influences the expression of ABC transporter genes. Eight major ABC transporter genes were selected for real-time PCR detection, including ABCB1, ABCC1, ABCG2 and so on. However, the results showed no significant changes between MFC cells transfected with miR-21 and the mock transfectants (Additional file 6: Figure S4), suggesting that miR-21 overexpression has no effect on ABC transporter genes in gastric cancer cells.

### Exosomal miR-21 regulates the PTEN/PI3K/AKT signaling pathway and enhances the anti-apoptotic ability of gastric cancer cells

**Fig. 6:**
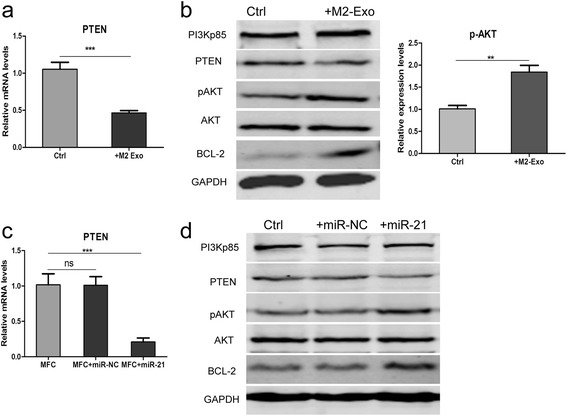
Exosomal transfer of miR-21 enhances gastric cancer cells chemoresistance via the PTEN/PI3K/AKT signaling pathway. **a** qRT-PCR detection of PTEN in MFC cells treated with or without M2-Exo. (*** *p* < 0.001). **b** Western blot assays for the expression of PI3Kp85, PTEN, p-AKT, AKT, BCL-2 in MFC cells treated with or without M2-Exo.GAPDH was used as the loading control. **c** qRT-PCR detection of PTEN in MFC cells transfected with miR-21 mimics or miR-NC or without transfection (ctrl). **d** Western blot assays for the expression of PI3Kp85, PTEN, p-AKT, AKT, BCL-2 in MFC cells transfected with miR-21 mimics or miR-NC or without transfection (ctrl). GAPDH was used as the loading control

Previous studies have showed that miR-21 could regulate tumor biological behavior through the PTEN/PI3K/AKT signaling pathway in several types of cancer, including gastric cancer. However, most of previous studies focused on the miR-21 expression level of cancer cells themselves. In our model, whether the exosomal transfer of functional miR-21 also has the same effect yet need further verification. So we examined the PI3K/AKT pathway related proteins in both M2-exos treated and miR-21 overexpression MFC cells. We found that the presence of M2-exos decreased the PTEN mRNA and protein expression level and increased AKT phosphorylation (Fig. 6a and b). Similar changes of MFC cells were observed after transfected with miR-21 (Fig. 6c and d). These data suggested that exosomal transfer of miR-21 enhances gastric cancer cells chemoresistance partially through the regulation of PTEN/PI3K/AKT signaling pathway.

In addition, there is accumulating evidence that resistant to apoptosis is responsible for drug resistance. Previous reports have revealed that Bcl-2, which was one of very important anti-apoptosis genes, was regulated by miR-21 directly [27]. So we next determined whether miR-21 affected Bcl-2 expression. Western blotting analyses indicated that M2-exos treatment and miR-21 overexpression dramatically increased the Bcl-2 protein level in MFC cells, which might be another mechanism responsible for the enhanced anti-apoptosis ability (Fig. 6b and d).

## Discussion

Cisplatin-based chemotherapy is now the most commonly used chemotherapeutic criterion in gastric cancer. Unfortunately, advanced GC patients who develop resistance to cisplatin have limited therapeutic options in the clinic at present [3]. Besides genetic changes of tumor cells themselves causing increased drug efflux or enhanced anti-apoptosis, drug resistance can result from the tumor microenvironment protecting tumor cells against treatment [28]. In the present study, we showed that exosomes derived from M2 macrophages express higher levels of miR-21 compared to unactivated macrophages, and that the exosomal transfer of miR-21 from M2 macrophages to gastric cancer cells could confer DDP resistance in these cells. The exosome-shuttled miR-21 promoted DDP resistance through the downregulation of PTEN, leading to a more active signaling through the PI3K/AKT pathway. In addition, M2-exos protected gastric cancer cells from chemotherapy induced apoptosis through the regulation of anti-apoptosis protein Bcl-2.

The role of exosomes in cancer as mediators of cell-cell communication within the microenvironment has gained increasing attention. Several studies have described the intriguing roles of exosomes in cancer progression through transfer a variety of proteins, DNA, and RNA [29]. Importantly, exosomes have been proved to be critically involved in the development of chemoresistance. Sousa et al. have demonstrated that exosomes from drug-resistant cancer cells can transfer the resistant phenotype to drug-sensitive cells, mainly through transferring of drug-efflux pumps and miRNAs [30]. Qu L et al. have also suggested that lncRNAs embedded in exosomes derived from sunitinib-resistant cells could confer the resistant phenotype to recipient RCC cells [31].

Apart from tumor cells, exosomes from tumor stromal cells also contribute to the acquisition of a resistant phenotype in cancer cells. Boelens et al. demonstrated that stromal cells orchestrate an intricate crosstalk with breast cancer cells by utilizing exosomes to regulate therapy resistance pathways [20]. Exosomes from cancer-associated fibroblast have been shown to promote proliferation and drug resistance of pancreatic cancer cells through transfer of chemoresistance-inducing factor snail [32]. A recent study reported that exosomal miR-21 can confer chemoresistance and an aggressive phenotype in ovarian cancer cells through its transfer from cancer-associated adipocytes and fibroblasts [24]. Similar to the above researches, here we demonstrated that exosomes from M2 polarized macrophages was sufficient to confer DDP resistance in gastric cancer cells both in vivo and in vitro; suggesting that tumor associated macrophages may support gastric cancer aggressiveness by secreting exosomes besides physical contact in the tumor microenvironment.

In recent years, it has become increasingly clear that miRNAs play an important role in the resistance of gastric cancer cells to chemotherapeutics [33, 34]. Importantly, exosome-mediated transfer of miRNAs within the tumor microenvironment has also been indicated as a significant mechanism for dissemination of drug resistance [23]. In this study, using miRNA microarray in M2 macrophage-derived exosomes, we found that miR-21 was the most abundant miRNA among the identified miRNAs. Furthermore, significantly higher miR-21 expression levels were detected in both M2 polarized macrophages and M2-exos than in unactivated macrophages by a qRT-PCR analysis. Next, we incubated gastric cancer cells with purified exosomes from M2 macrophages transfected with Cy3-labelled miR-21, and the transfer of miR-21 was confirmed by the detection of fluorescent signal in the gastric cancer cells using confocal microscopy.

Additionally, we explored the possible mechanism by which miR-21 may promote the DDP resistance in gastric cancer cells. Our data showed that miR-21 overexpression had no effect on ABC transporter genes in gastric cancer cells, one of the main mechanisms for chemoresistance. Previous studies have revealed that miR-21 expression was associated with resistance to a variety of chemotherapeutic agents in both solid and haematologic tumors. miR-21 promotes cell survival and confers resistance to cancer cells by regulating a set of tumor suppressor genes and apoptosis-associated genes [24, 35, 36]. Among these genes, PTEN/PI3K/AKT signaling pathway, which involved in cancer pathology and chemoresistance, has been shown to be managed by miR-21 in several types of cancer [37, 38]. Consistent with these findings, we demonstrated here that exosomal transfer of miR-21 led to down-regulation of PTEN and increased activation of AKT, resulting in more survival and less apoptosis in gastric cancer cells when treated with DDP. In addition, one important apoptosis-associated gene Bcl-2 was also elevated along with miR-21 overexpression, though the exact mechanism was not documented in our data. Moreover, the other potential miR-21 target genes may also be responsible for the effects of M2-exos on gastric cancer chemoresistance, which need further in-depth study.

## Conclusions

In summary, we demonstrate that M2 polarized macrophages confer DDP resistance in gastric cancer cells through exosomal transfer of functional miR-21. Our findings suggest that TAM derived exosomes are an important mediator for the reciprocity between TAM and gastric cancer cells, Moreover, we provide evidence to show that targeting exosomal miR-21 from TAM maybe a promising adjuvant therapeutic strategy for gastric cancer patients, especially cisplatin-resistant GC patients.

## Additional files

Additional file 1: Table S1. Antibody list for Western blot and Flow cytometry. (DOC 44 kb)

Additional file 2: Table S2. List of primers used for candidate genes. (DOC 37 kb)

Additional file 3: Figure S1. Co-cultivation with M2 polarized macrophages enhances the resistance of MGC-803 cells to cisplatin.a FACS analyses of CD68, CD80, CD163, and CD206 in Un-Mac or M2-polarized macrophages from human peripheral blood monocytes. b Cell viability assay of MGC-803 treated with various concentrations of DDP for 24 h, 48 h, and 72 h. c Flow cytometric analyses of apoptotic cells. MGC-803 cells were cultured alone or co-cultured with unactivated (Un-Mac) or M2 polarized macrophages, and then exposed to DDP for 48 h. The quantitative data are presented as the mean ± SD of triplicate experiments. (ns *p* > 0.05, ***p < 0.001). (TIF 2027 kb)

Additional file 4: Figure S2. M2-exos induce the resistance of MGC-803 cells to DDP in vitro.a Cell viability was assessed by CCK-8 assays. M2-derived conditioned medium (CM) or exosomes (M2-Exo) attenuated DDP-induced cell suppression. MGC-803 cells were pretreated with M2-derived CM or exosomes, and then exposed to DDP for 48 h. b Flow cytometric analyses of apoptotic cells. MGC-803 cells were exposed to DDP alone or DDP and M2-Exo for 48 h. The quantitative data are presented as the mean ± SD of triplicate experiments. (***p < 0.001). (TIF 739 kb)

Additional file 5: Figure S3. miR-21 enhances chemoresistance and decreases apoptosis in MGC-803 cells.aqRT-PCR detection of miR-21 in MFC and MGC-803 transfected with miR-21 mimics or miR-NC or without transfection (ctrl). (*** *p* < 0.001).b Cell viability assay of MGC-803 cells treated with DDP after transfected with miR-21 mimics or miR-NC or without transfection (ctrl). (ns*p* > 0.05,*** *p* < 0.001). c Cell apoptosis assay of MGC-803 cells treated with DDP after transfected with miR-21 mimics or miR-NC or without transfection (ctrl). The quantitative data are presented as the mean ± SD of triplicate experiments. (ns*p* > 0.05, ***p < 0.001). (TIF 1386 kb)

Additional file 6: Figure S4. qRT-PCR detection of ABCB1, ABCC1, ABCC2, ABCC3, ABCC4, ABCC5, ABCC6, ABCG2 mRNA expression in MFC cells transfected with miR-21 mimics or miR-NC or without transfection. GAPDH was used as an internal control. (TIF 660 kb)

## Abbreviations

ABC: ATP binding cassette
BMDM: Bone marrow–derived macrophage
CCK-8: Cell counting kit-8
DDP: Cisplatin
FACS: Fluorescence activated cell sorting
FBS: Fetal bovine serum
miR-21: microRNA-21
PBMC: Peripheral blood mononuclear cell
qRT-PCR: Quantitative Real-time PCR
TAM: Tumor associated macrophage

## References

[1] Torre LA, Bray F, Siegel RL, Ferlay J, Lortet-Tieulent J, Jemal A (2015). Global cancer statistics, 2012. CA Cancer J Clin.

[2] Chen W, Zheng R, Baade PD, Zhang S, Zeng H, Bray F, Jemal A, Yu XQ, He J (2016). Cancer statistics in China, 2015. CA Cancer J Clin.

[3] Orditura M, Galizia G, Sforza V, Gambardella V, Fabozzi A, Laterza MM, Andreozzi F, Ventriglia J, Savastano B, Mabilia A (2014). Treatment of gastric cancer. World J Gastroenterol.

[4] Gajewski TF, Schreiber H, Fu YX (2013). Innate and adaptive immune cells in the tumor microenvironment. Nat Immunol.

[5] Quail DF, Joyce JA (2013). Microenvironmental regulation of tumor progression and metastasis. Nat Med.

[6] Mantovani A, Allavena P, Sica A, Balkwill F (2008). Cancer-related inflammation. Nature.

[7] Noy R, Pollard Jeffrey W (2014). Tumor-associated macrophages: from mechanisms to therapy. Immunity.

[8] Qian BZ, Pollard JW (2010). Macrophage diversity enhances tumor progression and metastasis. Cell.

[9] Sica A, Mantovani A (2012). Macrophage plasticity and polarization: in vivo veritas. J Clin Invest.

[10] Mills CD (2012). M1 and M2 macrophages: oracles of health and disease. Crit Rev Immunol.

[11] Murray Peter J, Allen Judith E, Biswas Subhra K, Fisher Edward A, Gilroy Derek W, Goerdt S, Gordon S, Hamilton John A, Ivashkiv Lionel B, Lawrence T (2014). Macrophage activation and polarization: nomenclature and experimental guidelines. Immunity.

[12] Chen J, Yao Y, Gong C, Yu F, Su S, Chen J, Liu B, Deng H, Wang F, Lin L (2011). CCL18 from tumor-associated macrophages promotes breast cancer metastasis via PITPNM3. Cancer Cell.

[13] Cardoso AP, Pinto ML, Pinto AT, Oliveira MI, Pinto MT, Goncalves R, Relvas JB, Figueiredo C, Seruca R, Mantovani A (2014). Macrophages stimulate gastric and colorectal cancer invasion through EGFR Y(1086), c-Src, Erk1/2 and Akt phosphorylation and smallGTPase activity. Oncogene.

[14] Wu MH, Lee WJ, Hua KT, Kuo ML, Lin MT (2015). Macrophage infiltration induces gastric cancer invasiveness by activating the beta-catenin pathway. PLoS One.

[15] Ruffell B, Coussens LM (2015). Macrophages and therapeutic resistance in cancer. Cancer Cell.

[16] Mantovani A, Allavena P (2015). The interaction of anticancer therapies with tumor-associated macrophages. J Exp Med.

[17] Thery C (2011). Exosomes: secreted vesicles and intercellular communications. F1000 Biol Rep.

[18] Tkach M, Thery C (2016). Communication by extracellular vesicles: where we are and where we need to go. Cell.

[19] Penfornis P, Vallabhaneni KC, Whitt J, Pochampally R (2016). Extracellular vesicles as carriers of microRNA, proteins and lipids in tumor microenvironment. Int J Cancer.

[20] Boelens Mirjam C, Wu Tony J, Nabet Barzin Y, Xu B, Qiu Y, Yoon T, Azzam Diana J, Twyman-Saint Victor C, Wiemann Brianne Z, Ishwaran H (2014). Exosome transfer from stromal to breast cancer cells regulates therapy resistance pathways. Cell.

[21] Qu Z, Wu J, Wu J, Luo D, Jiang C, Ding Y (2016). Exosomes derived from HCC cells induce sorafenib resistance in hepatocellular carcinoma both in vivo and in vitro. J Exp Clin Cancer Res.

[22] Wang X, Xu C, Hua Y, Sun L, Cheng K, Jia Z, Han Y, Dong J, Cui Y, Yang Z (2016). Exosomes play an important role in the process of psoralen reverse multidrug resistance of breast cancer. J Exp Clin Cancer Res.

[23] Challagundla KB, Wise PM, Neviani P, Chava H, Murtadha M, Xu T, Kennedy R, Ivan C, Zhang X, Vannini I (2015). Exosome-mediated transfer of microRNAs within the tumor microenvironment and neuroblastoma resistance to chemotherapy. J Natl Cancer Inst.

[24] Au Yeung CL, Co N-N, Tsuruga T, Yeung T-L, Kwan S-Y, Leung CS, Li Y, Lu ES, Kwan K, Wong K-K (2016). Exosomal transfer of stroma-derived miR21 confers paclitaxel resistance in ovarian cancer cells through targeting APAF1. Nat Commun.

[25] Gordon S (2003). Alternative activation of macrophages. Nat Rev Immunol.

[26] Szakács G, Annereau J-P, Lababidi S, Shankavaram U, Arciello A, Bussey KJ, Reinhold W, Guo Y, Kruh GD, Reimers M (2004). Predicting drug sensitivity and resistance. Cancer Cell.

[27] Dong J, Zhao Y-P, Zhou L, Zhang T-P, Chen G (2011). Bcl-2 Upregulation induced by miR-21 via a direct interaction is associated with apoptosis and chemoresistance in MIA PaCa-2 pancreatic cancer cells. Arch Med Res.

[28] Klemm F, Joyce JA (2015). Microenvironmental regulation of therapeutic response in cancer. Trends Cell Biol.

[29] ELA S, Mager I, Breakefield XO, Wood MJ (2013). Extracellular vesicles: biology and emerging therapeutic opportunities. Nat Rev Drug Discov.

[30] Sousa D, Lima RT, Vasconcelos MH (2015). Intercellular transfer of cancer drug resistance traits by extracellular vesicles. Trends Mol Med.

[31] Qu L, Ding J, Chen C, Wu Z-J, Liu B, Gao Y, Chen W, Liu F, Sun W, Li X-F (2016). Exosome-transmitted lncARSR promotes sunitinib resistance in renal cancer by acting as a competing endogenous RNA. Cancer Cell.

[32] Richards KE, Zeleniak AE, Fishel ML, Wu J, Littlepage LE, Hill R (2017). Cancer-associated fibroblast exosomes regulate survival and proliferation of pancreatic cancer cells. Oncogene..

[33] Dehghanzadeh R, Jadidi-Niaragh F, Gharibi T, Yousefi M (2015). MicroRNA-induced drug resistance in gastric cancer. Biomed Pharmacother.

[34] Riquelme I, Letelier P, Riffo-Campos A, Brebi P, Roa J (2016). Emerging role of miRNAs in the drug resistance of gastric cancer. Int J Mol Sci.

[35] Giovannetti E, Erozenci A, Smit J, Danesi R, Peters GJ (2012). Molecular mechanisms underlying the role of microRNAs (miRNAs) in anticancer drug resistance and implications for clinical practice. Crit Rev Oncol Hematol.

[36] Shi GH, Ye DW, Yao XD, Zhang SL, Dai B, Zhang HL, Shen YJ, Zhu Y, Zhu YP, Xiao WJ, Ma CG (2010). Involvement of microRNA-21 in mediating chemo-resistance to docetaxel in androgen-independent prostate cancer PC3 cells. Acta Pharmacol Sin.

[37] Xiong B (2013). MiR-21 regulates biological behavior through the PTEN/PI-3 K/Akt signaling pathway in human colorectal cancer cells. Int J Oncol..

[38] Yang S-m, Huang C, Li X-f, Yu M-z, He Y, Li J (2013). miR-21 confers cisplatin resistance in gastric cancer cells by regulating PTEN. Toxicology.

