# Supplementary material for: Exosomal transfer of tumor-associated macrophage-derived miR-21 confers cisplatin resistance in gastric cancer cells
Source: J Exp Clin Cancer Res. 2017 Apr 13;36:53. doi: 10.1186/s13046-017-0528-y (PMC5390430; doi:10.1186/s13046-017-0528-y)
Supplement: Supplementary file 1 — Antibody list for Western blot and Flow cytometry. (DOC 44 kb) [file 13046_2017_528_MOESM1_ESM.doc]

**Table S1: Antibody list for Western blotand Flow cytometry.**

| **Name** | **Host** | **Clone** | **Company** | **Dilution** |
| --- | --- | --- | --- | --- |
| CD68-PE | Mouse | Y1/82A | BD Pharmingen | 1:50(FC) |
| CD163-PE | Mouse | GHI/61 | BD Pharmingen | 1:50(FC) |
| CD206-APC | Mouse | 19.2 | eBioscience | 1:50(FC) |
| CD206-APC | Rat | C068C2 | Biolegend | 1:50(FC) |
| CD80-PCy7 | Mouse | L307.4 | BD Pharmingen | 1:50(FC) |
| F4/80-PE | Rat | BM8 | eBioscience | 1:50(FC) |
| CD11b-PCy5.5 | Rat | M1/70 | BD Pharmingen | 1:50(FC) |
| CD86-V450 | Rat | GL1 | BD Pharmingen | 1:50(FC) |
| CD63 | Rabbit | N/A | SBI | 1:1000(WB) |
| CD9 | Rabbit | N/A | SBI | 1:1000(WB) |
| CD81 | Rabbit | N/A | SBI | 1:1000(WB) |
| HSP70 | Rabbit | N/A | SBI | 1:1000(WB) |
| PI3K p85 | Rabbit | 19H8 | Cell Signaling | 1:1000(WB) |
| p-AKT(Thr308) | Rabbit | D25E6 | Cell Signaling | 1:1000(WB) |
| AKT | Rabbit | 11E7 | Cell Signaling | 1:1000(WB) |
| PTEN | Rabbit | D5G7 | Cell Signaling | 1:1000(WB) |
| BCL-2 | Rabbit | N/A | Beyotime | 1:1000(WB) |
| GAPDH | Mouse | N/A | Beyotime | 1:1000(WB) |
