# Supplementary material for: Exosomal transfer of tumor-associated macrophage-derived miR-21 confers cisplatin resistance in gastric cancer cells
Source: J Exp Clin Cancer Res. 2017 Apr 13;36:53. doi: 10.1186/s13046-017-0528-y (PMC5390430; doi:10.1186/s13046-017-0528-y)
Supplement: Supplementary file 2 — List of primers used for candidate genes. (DOC 37 kb) [file 13046_2017_528_MOESM2_ESM.doc]

**Table S2. List of primers used for candidate genes.**

| **Gene** | **FORWARD** | **REVERSE** |
| --- | --- | --- |
| ARG1 | 5-CAGAAGAATGGAAGAGTCAG | 5-CAGATATGCAGGGAGTCACC |
| INOS | 5-CTGCAGCACTTGGATCAGGAAC | 5-GGAGTAGCCTGTGTGCACCT |
| TGF-β | 5-ATTCCTGGCGTTACCTTGG | 5-AGCCCTGTATTCCGTCTCCT |
| IL-10 | 5-GCCTTATCGGAAATGATCCA | 5-TGAGGGTCTTCAGCTTCTCAC |
| PTEN | 5-AGCCTCTTGATGTGTGCATT | 5-CCATTGGTAGCCAAACGGAAC |
| ABCB1 | 5-GCGCAGAAGGGCATCTACTT | 5-ACCTTTAACAGCCCTTGCCT |
| ABCC1 | 5-GAGGCATCTCAGCAACTCGT | 5-CCACGTCTCCTCCTTAGCTC |
| ABCC2 | 5-CTTCCCTTGAGGCAGATGGT | 5-CCCAAGGGAATCCACACAAGA |
| ABCC3 | 5-TGAGGATGCGGTCCTACTGA | 5-GGCCGTGGGGTCAGAAATAA |
| ABCC4 | 5-CACACCGAGGTGAAACCCAA | 5-CCTCTCCGAGGTGCTTTGAG |
| ABCC5 | 5-CTAGCTGGTCGTTTCACGGT | 5-CCCTGAGAATTCACGGTGCT |
| ABCC6 | 5-TGGAGACAGTGCAGCTCAAG | 5-CATCTGCATCTCCGTCCCTG |
| ABCG2 | 5-CCTTGCCAGATAAGAGGGGT | 5-TGCCTCAAGAGACAGGCAAA |
| GAPDH | 5-AGGAGAGTGTTTCCTCGTCC | 5-GGCCTCACCCCATTTGATGT |
