# Supplementary figures and images for: Exosomal transfer of tumor-associated macrophage-derived miR-21 confers cisplatin resistance in gastric cancer cells
Source: J Exp Clin Cancer Res. 2017 Apr 13;36:53. doi: 10.1186/s13046-017-0528-y (PMC5390430; doi:10.1186/s13046-017-0528-y)

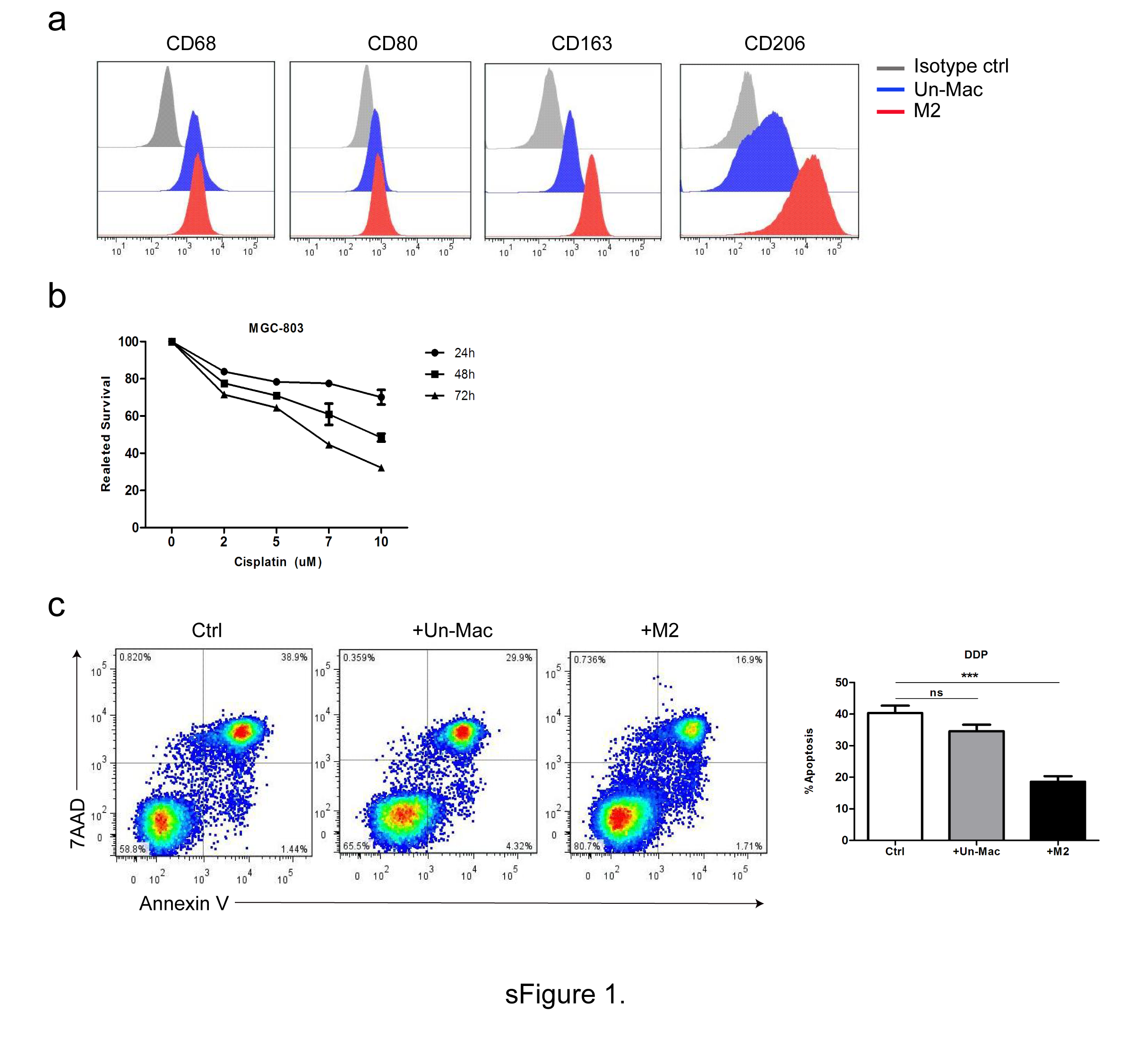

Supplement: Supplementary file 3 — Co-cultivation with M2 polarized macrophages enhances the resistance of MGC-803 cells to cisplatin.a FACS analyses of CD68, CD80, CD163, and CD206 in Un-Mac or M2-polarized macrophages from human peripheral blood monocytes. b Cell viability assay of MGC-803 treated with various concentrations of DDP for 24 h, 48 h, and 72 h. c Flow cytometric analyses of apoptotic cells. MGC-803 cells were cultured alone or co-cultured with unactivated (Un-Mac) or M2 polarized macrophages, and then exposed to DDP for 48 h. The quantitative data are presented as the mean ± SD of triplicate experiments. (ns p > 0.05, ***p < 0.001). (TIF 2027 kb) [file 13046_2017_528_MOESM3_ESM.tif]

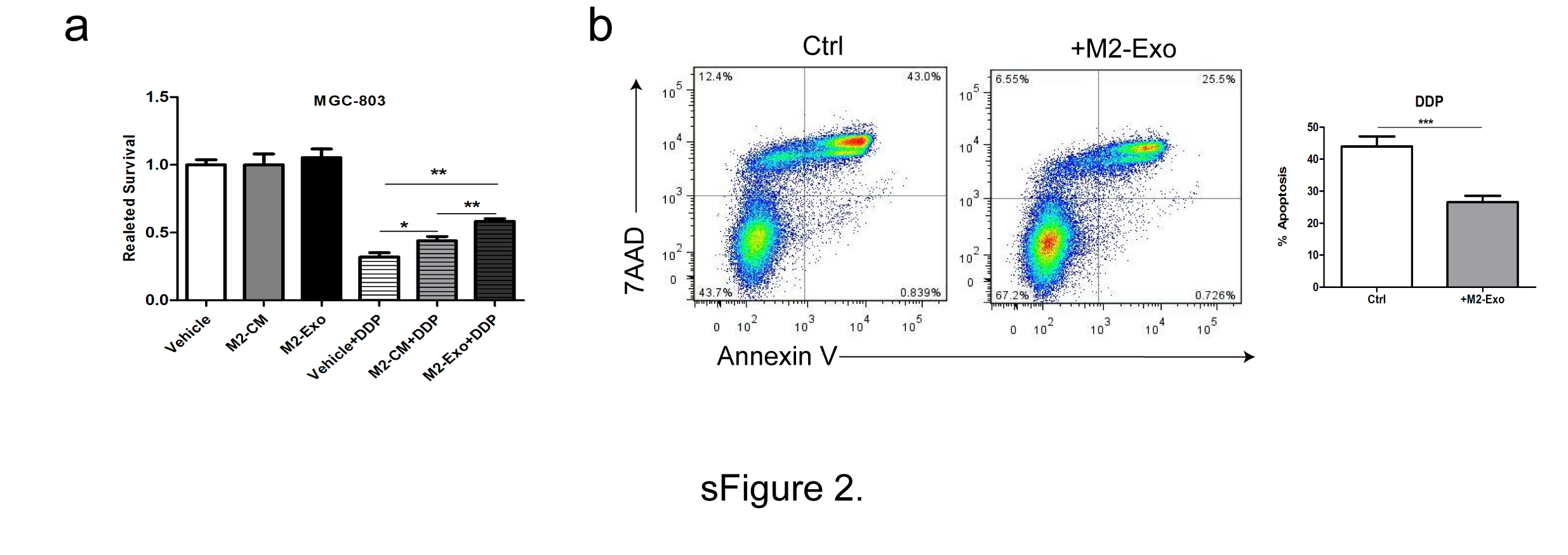

Supplement: Supplementary file 4 — M2-exos induce the resistance of MGC-803 cells to DDP in vitro.a Cell viability was assessed by CCK-8 assays. M2-derived conditioned medium (CM) or exosomes (M2-Exo) attenuated DDP-induced cell suppression. MGC-803 cells were pretreated with M2-derived CM or exosomes, and then exposed to DDP for 48 h. b Flow cytometric analyses of apoptotic cells. MGC-803 cells were exposed to DDP alone or DDP and M2-Exo for 48 h. The quantitative data are presented as the mean ± SD of triplicate experiments. (***p < 0.001). (TIF 739 kb) [file 13046_2017_528_MOESM4_ESM.tif]

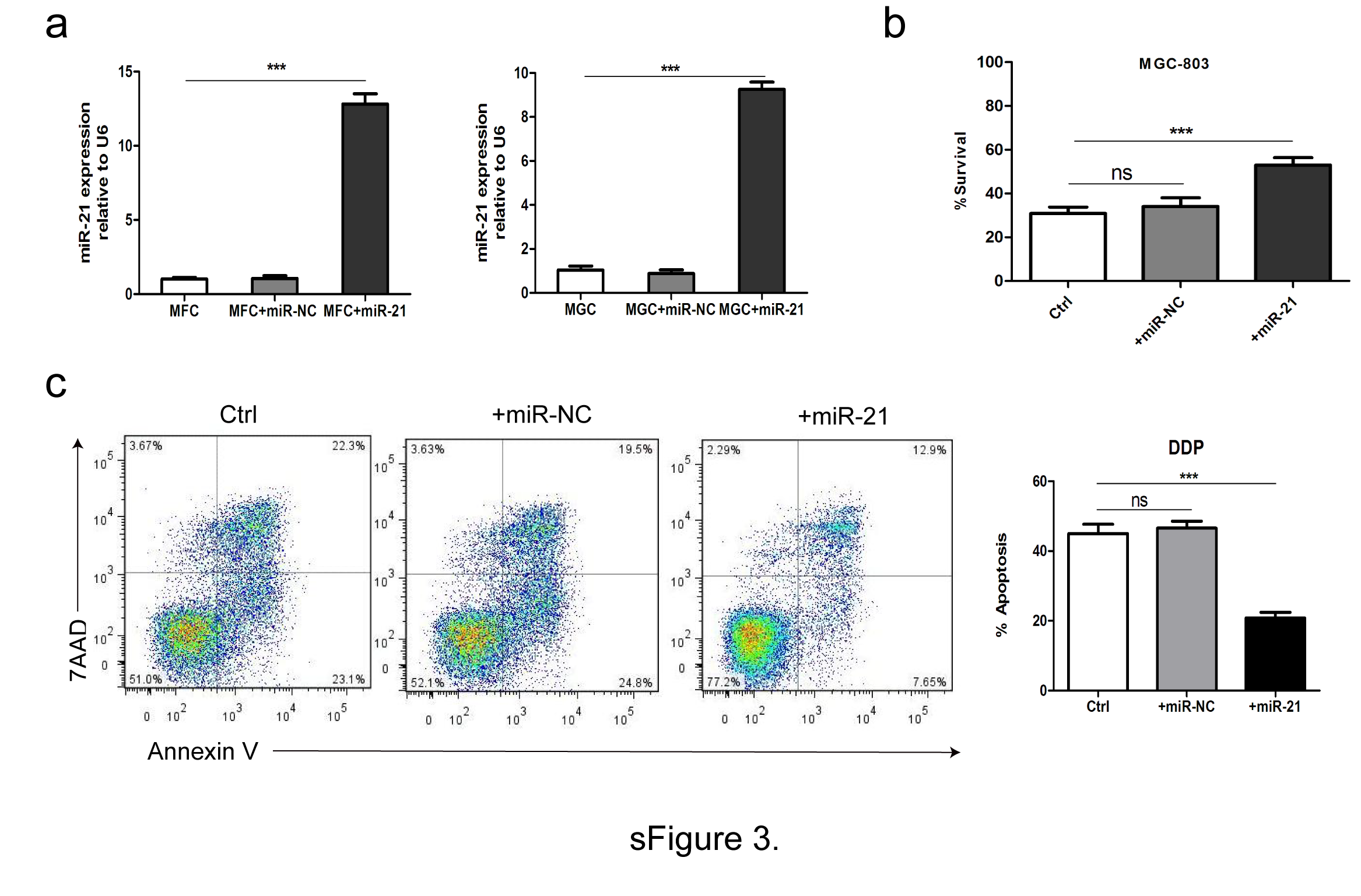

Supplement: Supplementary file 5 — miR-21 enhances chemoresistance and decreases apoptosis in MGC-803 cells.aqRT-PCR detection of miR-21 in MFC and MGC-803 transfected with miR-21 mimics or miR-NC or without transfection (ctrl). (*** p < 0.001).b Cell viability assay of MGC-803 cells treated with DDP after transfected with miR-21 mimics or miR-NC or without transfection (ctrl). (nsp > 0.05,*** p < 0.001). c Cell apoptosis assay of MGC-803 cells treated with DDP after transfected with miR-21 mimics or miR-NC or without transfection (ctrl). The quantitative data are presented as the mean ± SD of triplicate experiments. (nsp > 0.05, ***p < 0.001). (TIF 1386 kb) [file 13046_2017_528_MOESM5_ESM.tif]

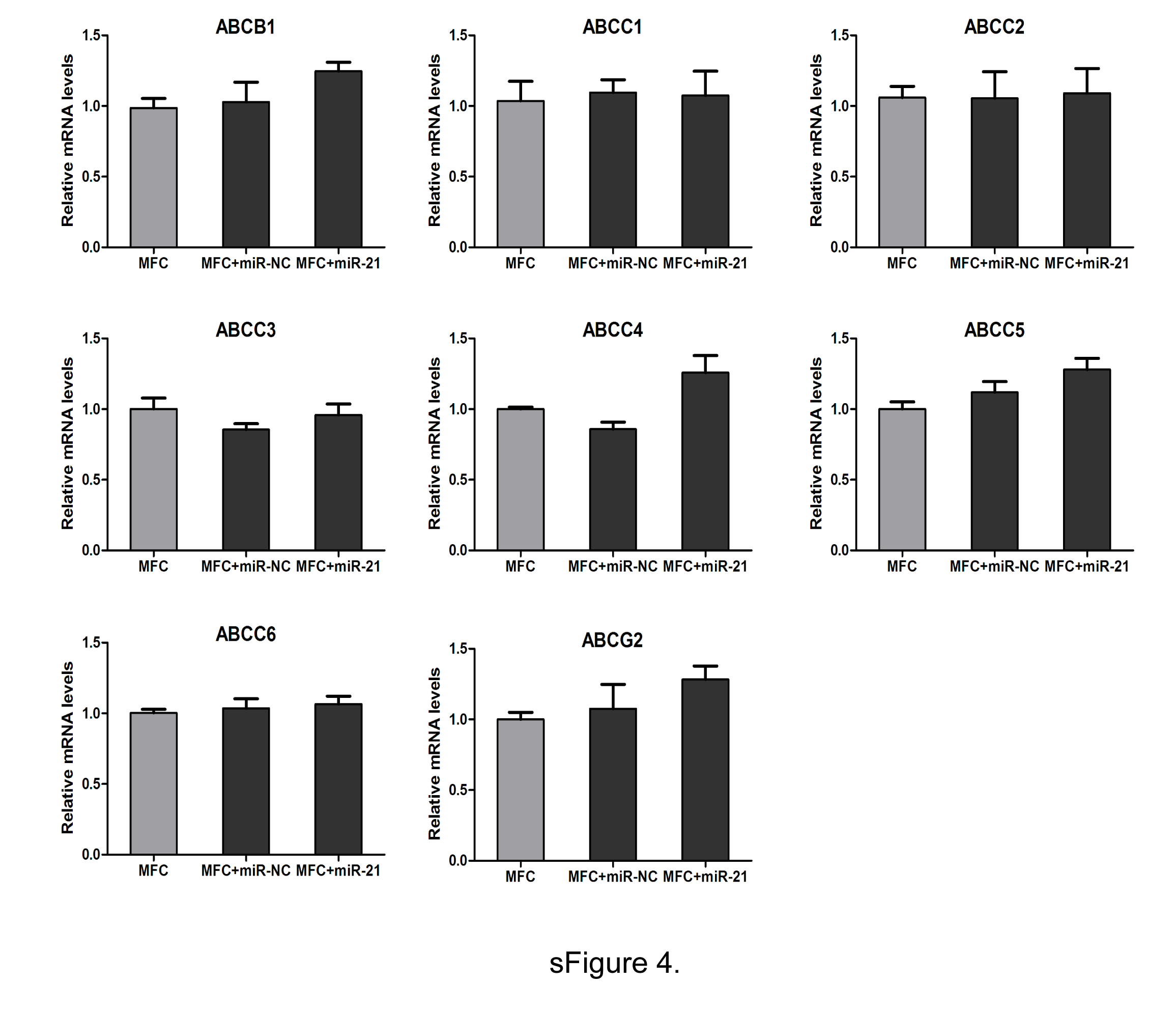

Supplement: Supplementary file 6 — qRT-PCR detection of ABCB1, ABCC1, ABCC2, ABCC3, ABCC4, ABCC5, ABCC6, ABCG2 mRNA expression in MFC cells transfected with miR-21 mimics or miR-NC or without transfection. GAPDH was used as an internal control. (TIF 660 kb) [file 13046_2017_528_MOESM6_ESM.tif]
